# Antibacterial and Antibiofilm Activities of Makaluvamine Analogs

**DOI:** 10.3390/microorganisms2030128

**Published:** 2014-09-12

**Authors:** Bhavitavya Nijampatnam, Dwayaja H. Nadkarni, Hui Wu, Sadanandan E. Velu

**Affiliations:** 1 Department of Chemistry, University of Alabama at Birmingham, 901, 14th Street South, Birmingham, AL 35294-1240, USA; E-Mails: snij@uab.edu (B.N.); dwayaja@uab.edu (D.H.N.); 2 Department of Pediatric Dentistry, UAB School of Dentistry, 1919 7th Avenue South, Birmingham, AL 35294-0007, USA; E-Mail: hwu@uab.edu

**Keywords:** makaluvamine, pyrroloiminoquinone, marine alkaloid, *Streptococcus mutans*, biofilm, antibacterial, dental caries

## Abstract

*Streptococcus mutans* is a key etiological agent in the formation of dental caries. The major virulence factor is its ability to form biofilms. Inhibition of *S. mutans* biofilms offers therapeutic prospects for the treatment and the prevention of dental caries. In this study, 14 analogs of makaluvamine, a marine alkaloid, were evaluated for their antibacterial activity against *S. mutans* and for their ability to inhibit *S. mutans* biofilm formation. All analogs contained the tricyclic pyrroloiminoquinone core of makaluvamines. The structural variations of the analogs are on the amino substituents at the 7-position of the ring and the inclusion of a tosyl group on the pyrrole ring N of the makaluvamine core. The makaluvamine analogs displayed biofilm inhibition with IC_50_ values ranging from 0.4 μM to 88 μM. Further, the observed bactericidal activity of the majority of the analogs was found to be consistent with the anti-biofilm activity, leading to the conclusion that the anti-biofilm activity of these analogs stems from their ability to kill *S. mutans*. However, three of the most potent *N*-tosyl analogs showed biofilm IC_50_ values at least an order of magnitude lower than that of bactericidal activity, indicating that the biofilm activity of these analogs is more selective and perhaps independent of bactericidal activity.

## 1. Introduction

Bacterial biofilms are a facet of our daily life [1]. We encounter them in various forms, such as the residue that clogs drains, the slime that coats rocks near a stream and the plaque that forms on the surfaces of our teeth [2,3,4]. Bacterial biofilms are defined as surface-attached bacterial communities embedded in an extracellular matrix composed of polysaccharides [4]. When compared to planktonic bacteria, their free-living counterparts, biofilms offer increased resistance to host immune responses, antibiotics and biocides [5]. Seventy-five percent of all infections have been implicated to the existence of biofilms, including urinary tract infections, catheter infections, middle-ear infections, infective endocarditis and dental caries [6].

Dental caries is classified as one of the most common chronic infectious diseases. It is associated with a substantial economic burden worldwide [7,8]. It is defined as an infection of bacterial origin that causes demineralization of the hard surfaces of the teeth [9]. If left untreated, the infection results in severe pain, bacteremia and subsequent tooth loss [10]. Though individuals remain susceptible to the disease throughout their lifetime, according to the most recent National Health and Nutrition Examination Survey, 42% of children aged two to 11 have had dental caries in their primary teeth, and by adulthood, 92% have experienced some decay in their permanent dentition [10].

Recent analyses with ribosomal RNA-based technologies have been employed to discover both the diversity of dental biofilms and their role in the development of dental carries [11]. During the early stages of biofilm formation, planktonic bacterial cells either directly attach to surfaces of the oral cavity or indirectly bind to other bacterial cells that have already colonized [12]. The oral bacteria interact cooperatively with other species and form a complex multispecies ecosystem. Within this ecosystem, pivotal regulatory factors, such as metabolic communication, genetic exchange, production of inhibitory factors and quorum-sensing, all contribute to the bacterial composition of biofilms [11].

Although different bacteria have been found to be associated with the pathogenesis of dental caries, substantial evidence indicates a causative relationship between dental caries and the mutans streptococcal group represented by *Streptococcus mutans* [13]. Various studies demonstrate that the development of caries is preceded by increased colonization with the *S. mutans* [14]. The bacterium has been isolated from 95% of children with dental caries and was found to comprise up to 30%–50% of the plaque microbiota in carious lesions [15]. In contrast, only 1% of the oral microbiota was found to comprise *S. mutans* in caries-free children [16]. The ability of *S. mutans* to adhere to the tooth surface and to incorporate into dental biofilms is paramount in the development and progression of the disease and is therefore designated as the primary etiological agent associated with the initiation and progression of dental caries [17].

Several strategies have been attempted in the prevention of dental caries, including decreasing the antimicrobial activity [18], replacement of sucrose with other sweeteners [19,20] and the inhibition of glucosyltransferases that are crucial for the biosynthesis of the biofilm matrix by either the vaccine approach [21] or enzymatic inhibitors [22]. However, limited success has been achieved in terms of the effectiveness and economic feasibility of these treatment strategies. Since *S. mutans* plays a precarious role in the pathogenesis of dental caries, it represents an important target for therapeutic interventions [23]. It is believed that decreasing the population of the cariogenic bacterium and preventing the ability of *S. mutans* to form a biofilm is essential in devising therapeutic and preventive strategies for dental caries [24].

**Figure 1:**
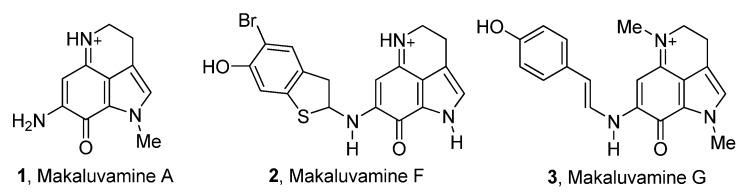
A few examples of naturally occurring makaluvamines.

Our lab has a longstanding interest in the synthesis and biological evaluation of marine alkaloids. In our efforts to identify inhibitors of *S. mutans* biofilms, we investigated a class of marine alkaloids, called makaluvamines, for its ability to inhibit *S. mutans* and its biofilm. Makaluvamines A–P are a group of 16 marine alkaloids isolated mainly from four species of marine sponges: namely the Fijian sponge, *Zyzzya* cf. *marsailis* [25], the Indonesian sponge, *Histodermella* sp*.* [26], the Pohnpeian sponge, *Zyzzya fuliginosa* [27], and the Jamaican sponge, *Smenospongia aurea* [28]*.* A few examples of makaluvamines are given in Figure 1.

We have explored this class of compounds extensively for their anticancer activity and successfully identified preclinical candidates in the past [29,30]. These compounds were thought to mainly act as DNA topoisomerase II inhibitors, until recently. Makaluvamine analogs have now been shown to inhibit cell proliferation and cell cycle progression, to induce apoptosis and to modulate the expression of several genes, such as MDM2, p21 and p53 [31]. As a part of our research on identifying drug leads from marine natural products, we were interested in evaluating the antibacterial and anti-biofilm activities of the analogs of marine alkaloid makaluvamine.

## 2. Experimental Section

### 2.1. Synthesis of Makaluvamine Analogs

#### 2.1.1. General Considerations

Chemical reactions were monitored with thin layer chromatography (TLC) using silica gel, UV254, 250 μm plates with a fluorescent indicator (Dynamic Absorbent, Inc., Atlanta, GA, USA). The TLC spots were observed under UV light with wavelengths of 254 nm and 365 nm. The reaction mixtures were purified by column chromatography using silica gel (32–63 μm) (Dynamic Absorbent, Inc., Atlanta, GA, USA). The amount (weight) of silica gel for column chromatography was in the range of 50–100-times the amount of the crude compounds being separated. Proton nuclear magnetic resonance (^1^H–NMR) and carbon nuclear magnetic resonance (^13^C–NMR) spectra were recorded on Bruker DPX 300 and DRX 400 spectrometers (Billerica, MA, USA) using TMS or appropriate solvent signals as the internal standard. The values of chemical shift are given in parts per million (ppm) relative to tetramethylsilane and coupling constants (*J*) in Hz. Mass spectra were recorded on an Applied Biosystems 4000 Q Trap instrument. Anhydrous solvents used for reactions were purchased in Sure-Seal™ bottles from Aldrich Chemical Company (St. Louis, MO, USA). Other reagents were purchased from Aldrich (St. Louis, MO, USA), Lancaster (Ward Hill, MA, USA) or Fisher chemical companies (Suwanee, GA, USA) and used as received.

#### 2.1.2. General Procedure for Amination

Compound **4** (1 equivalent) was dissolved in anhydrous MeOH (25 mL), and a solution of amine (**5a**–**d**) (1.2 equivalent) in anhydrous MeOH (5 mL) was added drop-wise. The resulting solution was stirred at room temperature. The reaction was completed in 20 h as indicated by TLC analysis in MeOH/CHCl_3_ (1:20). The reaction mixture was quenched by adding TFA (2 equivalent) and stirred for 15 min. The solvent was removed under reduced pressure, co-evaporated with CHCl_3_ to remove excess TFA, and the residue obtained was purified by flash column chromatography on Si gel using MeOH:CHCl_3_ (1/40) as the eluent to furnish Compounds **6a**–**d**. *N*-tosyl makaluvamine analogs **6a**–**d** were then subjected to detosylation, as described below, without further characterization.

#### 2.1.3. General Procedure for Detosylation Using NaOMe

*N*-tosyl makaluvamine analog **6a**–**d** (1 equivalent) was dissolved in anhydrous MeOH containing NaOMe (10 equivalent), and the reaction mixture was stirred for 45 min at room temperature. TLC analysis in MeOH/CHCl_3_ (1:20) revealed that the reaction was complete. The reaction mixture was cooled to 0 °C and quenched with TFA (30 equivalent). It was stirred further at room temperature for 30 min, and the solvent was removed under reduced pressure to obtain the crude product. The crude product was then purified by flash column chromatography over Si gel using MeOH:CHCl_3_ (1/20) as the eluent to obtain the pure detosylated makaluvamines **1a**–**d** (42%–48% yield over two steps from Compound **4**).

7-(4-Dimethylamino)benzylamino-3,4-dihydro-pyrrolo[4,3,2-*de*]quinolin-8(1H)-one trifluoroacetate salt (**1a**): Compound **1a** was prepared by following the general procedure, starting from Compound **4** (0.100 g, 0.21 mmol) in MeOH (25 mL) and 4-dimethylaminobenzylamine (0.038 g, 0.26 mmol) to obtain the tosyl intermediate, which was detosylated using NaOMe (0.091 g, 1.70 mmol) in MeOH. (0.039 g, 42%); ^1^H NMR (CD_3_OD) δ 2.93 (s, 6H, N-CH_3_), 2.96 (t, 2H, *J* = 7.6 Hz, CH_2_), 3.83 (t, 2H, *J* = 7.6 Hz, N–CH_2_), 4.48 (s, 2H, benzyl CH_2_), 5.46 (s, 1H, quinone ring CH), 6.78 (d, 2H, *J* = 8.4 Hz, benzene ring CH), 7.15 (s, 1H, pyrrole ring CH), 7.19 (d, 2H, *J* = 8.4 Hz, benzene ring CH); ^13^C NMR (CD_3_OD) δ 19.5, 41.0, 44.2, 47.9, 86.2, 114.3, 120.2, 123.9, 124.9, 125.6, 127.1, 129.5, 151.9, 155.0, 159.7 and 168.9; MS (ES^+^) *m/z* 321 (M^+^).

7-(4-Isopropylbenzylamino)-3,4-dihydro-pyrrolo[4,3,2-*de*]quinolin-8(1H)-one trifluoroacetate salt (**1b**): Compound **1b** was prepared by following the general procedure, starting from Compound **4** (0.100 g, 0.21 mmol) in MeOH (15 mL) and 4-isopropylbenzylamine (0.038 g, 0.26 mmol) to obtain the tosyl intermediate, which was detosylated using NaOMe (0.091 g, 1.70 mmol) in MeOH. (0.041 g, 44%); ^1^H NMR (CD_3_OD) δ 1.23 (d, 6H, *J* = 8.7 Hz, CH_3_), 2.89 (septet, 1H, *J* = 8.7 Hz, CH), 2.95 (t, 2H, *J* = 7.6 Hz, CH_2_ ), 3.83 (t, 2H, *J* = 7.6 Hz, N–CH_2_), 4.56 (s, 2H, benzyl CH_2_), 5.41 (s, 1H, quinone ring CH), 7.15 (s, 1H, pyrrole ring CH), 7.25 (s, 4H, benzene ring CH); ^13^C NMR (CD_3_OD) δ 19.5, 24.4, 35.1, 44.2, 47.9, 86.4, 120.2, 123.8, 125.7, 127.1, 128.0, 128.4, 134.5, 150.0, 155.2, 159.9 and 168.8; MS (ES^+^) *m/z* 320 (M^+^).

7-[(Thiophen-2-yl)methylamino]-3,4-dihydro-pyrrolo[4,3,2-*de*]quinolin-8(1H)-one trifluoroacetate salt (**1c**): Compound **1c** was prepared by following the general procedure, starting from Compound **4** (0.100 g, 0.21 mmol) in MeOH (25 mL) and thiophene-2-methylamine (0.036 g, 0.26 mmol) to obtain the tosyl intermediate, which was detosylated using NaOMe (0.113 g, 2.10 mmol) in MeOH. (0.041 g, 48%); ^1^H NMR (CD_3_OD) δ 2.95 (t, 2H, *J* = 7.6 Hz, CH_2_), 3.85 (t, 2H, *J* = 7.6 Hz, N-CH_2_), 4.76 (s, 2H, CH_2_), 5.56 (s, 1H, quinone ring CH), 6.99 (dd, 1H, *J*_1_ *=* 5.1 Hz , *J*_2_ *=* 1.2 Hz, thiophene ring CH), 7.10 (d, 1H, *J* *=* 2.7 Hz, thiophene ring CH), 7.14 (s, 1H, pyrrole ring CH), 7.37 (dd, 1H, *J*_1_ *=* 5.1 Hz, *J*_2_ *=* 1.2 Hz, pyrrole ring CH). ^13^C NMR (CD_3_OD) δ 19.4, 43.0, 44.4, 86.5, 120.2, 123.6, 125.7, 126.9, 127.1, 127.9, 128.0, 139.6, 154.6, 160.1 and 168.8. MS (ES^+^) *m/z* 284 (M^+^).

7-[(Furan-2-yl)methylamino]-3,4-dihydro-pyrrolo[4,3,2-*de*]quinolin-8(1H)-one trifluoroacetate salt (**1d**): Compound **1d** was prepared by following the general procedure, starting from Compound **4** (0.100 g, 0.21 mmol) in MeOH (25 mL) and furfuryl amine (0.031 g, 0.26 mmol) to obtain the tosyl intermediate, which was detosylated using NaOMe (0.081 g, 1.49 mmol) in MeOH. (0.035 g, 43%); ^1^H NMR (CD_3_OD) δ 2.97 (t, 2H, *J* = 7.5 Hz, CH_2_), 3.88 (t, 2H, *J* = 7.5 Hz, N-CH_2_), 4.58 (s, 2H, CH_2_), 5.63 (s, 1H, quinone ring CH), 6.40 (s, 1H, furan ring CH), 6.41 (s, 1H, furan ring CH), 7.16 (s, 1H, pyrrole ring CH), 7.50 (t, 1H, *J* = 1.2 Hz, furan ring CH); ^13^C NMR (CD_3_OD) δ 19.5, 41.1, 44.4, 86.3, 109.9, 111.6, 120.2, 123.6, 125.7, 127.1, 144.2, 150.3, 155.0, 160.2 and 168.8; MS (ES^+^) *m/z* 268 (M^+^).

### 2.2. Biological Assays

#### 2.2.1. Bacterial Strains, Culture Conditions and Chemicals

*S. mutans* UA159 was grown statically and aerobically at 37 °C with 5% CO_2_ in Todd-Hewitt broth, or a THB agar plate, or chemically-defined biofilm medium (CDBM) supplemented with 1% sucrose.

#### 2.2.2. *S. Mutans* Biofilm Inhibition Assay

A single colony of *S. mutans* was inoculated into 3 mL THB and incubated for 24 h. The overnight cultures were then inoculated at 1:100 dilutions into fresh THB to allow bacteria to grow until they reached the exponential growth phase at an optical density at 470 nm (OD_470_) of 0.6 measured by the microplate reader (BioTekELx, Winooski, VT, USA). The exponentially-grown bacteria were then inoculated at 1:100 dilutions with CDBM containing 1% sucrose for biofilm assays. Compounds at the indicated concentrations were added into the inoculated bacterial broth and incubated for 16 h. For control cultures, the corresponding volume of DMSO was added. The final concentration of DMSO was 1% in each well. After 16 h, planktonic cells were washed with water three times, and the remaining biofilms were stained with 0.1% crystal violet. The concentration of crystal violet was measured at OD562. Each assay was done with duplicate samples and replicated three times. The concentration of the potent compounds that inhibited *S. mutans* biofilm formation by 50% (IC_50_) was determined by serial dilutions.

#### 2.2.3. *S. Mutans* Growth Inhibition Assay

The exponentially grown bacteria harvested at OD_470_ of 0.6 were inoculated at 1:100 dilutions in the presence of indicated compound in THB. Growth was monitored at OD_470_ after 6 h. For control cultures, the corresponding volume of DMSO was added. The final concentration of DMSO was 1% in each well. Each assay was done with duplicate samples and replicated three times. The concentration of the potent compounds that inhibited *S. mutans* growth by 50% (MIC_50_) was determined by serial dilutions.

## 3. Results and Discussion

### 3.1. Synthesis of Makaluvamine Analogs

**Figure 2:**
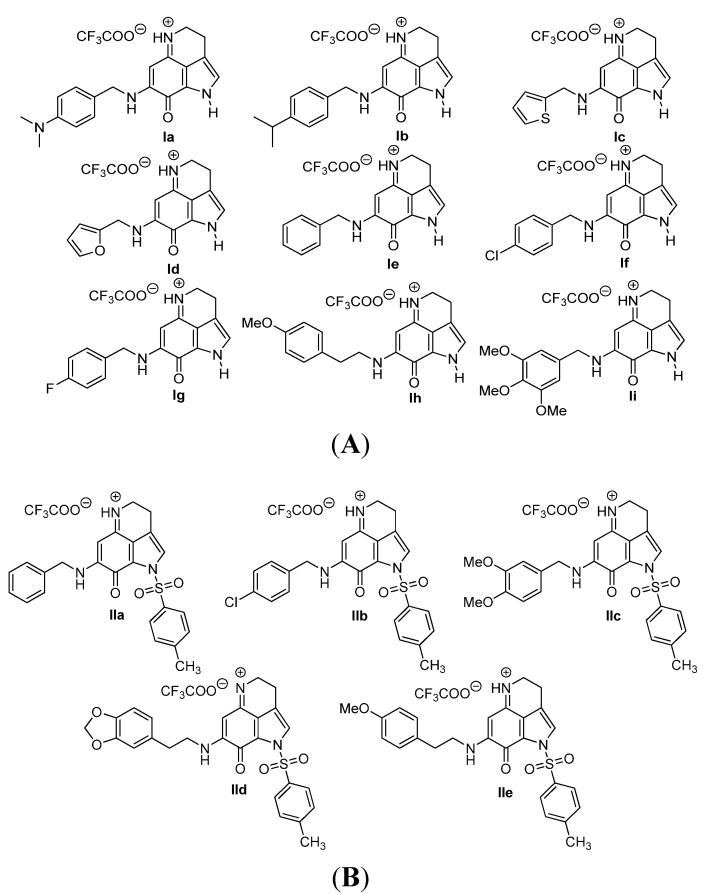
(**A**) Makaluvamine analogs with free pyrrole NH (Class I); (**B**) makaluvamine analogs with a tosyl group on pyrrole N (Class II).

We have evaluated 14 analogs of makaluvamines (Figure 2A,B) against *S. mutans* for their bactericidal and anti-biofilm activity. All analogs contained the tricyclic pyrroloiminoquinone core. The structural variations occur at the amino substituents at the 7-position of the ring. These substituents were constituted of structurally diverse amino substituents. This included a variety of substituted benzyl amino, phenethyl amino, thiophene methyl amino and furan methyl amino groups. Compounds were categorized in two classes: analogs with free pyrrole NH (Class I) (Figure 2A) and analogs with a tosyl group on pyrrole N (Class II) (Figure 2B).

**Scheme 1:**
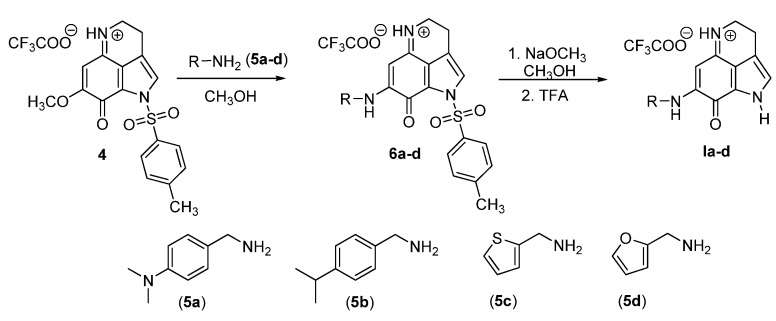
Synthesis of makaluvamine analogs **Ia**–**d**.

The synthesis of ten analogs (**Ie**–**i** and **IIa**–**e**) has already been reported from our laboratory in the publications related to their anticancer activities [29,30]. Four new analogs (**Ia**–**d**) were synthesized following the literature procedure as outlined in Scheme 1. They were synthesized in two steps starting from the known tricyclic pyrroloiminoquinone compound, **4**. We prepared this Compound **4** following the 4,6,7-trimethoxyindole approach described previously [32]. Treatment of Compound **4** with amine derivatives (**5a**–**d**) in anhydrous MeOH at room temperature provided the aminated Compounds **6a**–**d**. Intermediate products **6a**–**d** were subjected to detosylation reaction without further purification and characterization. Removal of the tosyl protecting group from the Compound **6a**–**d** was accomplished by treatment with NaOMe in MeOH to obtain the final products, **Ia**–**d**, in a 42%–48% yield. All final products were completely characterized.

### 3.2. Biological Evaluation

The synthesized makaluvamine analogs were first evaluated for their biofilm inhibitory activity using a previously reported assay [33]. The results are summarized in Table 1. Interestingly, all makaluvamine analogs showed biofilm inhibition with the activities ranging from 0.4 μM to 88 μM. Our studies show that compounds in Class II are more potent when compared to Class I compounds. The most active compound from these studies is the methylenedioxy phenethyl analog, which contained a tosyl group on the pyrrole N (Compound **Id**, Entry 13, 0.4 μM). This compound was found to be more potent compared to the previously reported most active marine analog of the 2-aminoimidazole scaffold (Compound **2A4**, Entry 15, 0.94 μM) [34]. We suspect that the presence of the tosyl group makes a significant contribution to the activity of the compound in inhibiting *S. mutans* biofilm formation. For instance, when comparing Compounds **Ih** and **IIe**, where the only structural difference is seen in the presence of the tosyl group, the IC_50_ values differ from 70.1 μM (Entry 8) to 2.0 μM (Entry 14). In this particular example, the tosyl group amplifies the activity by an order of magnitude. A similar account can be seen when comparing Compounds **If** and **IIb**, whose activities range 13.9 (Entry 6) and 2.5 μM (Entry 11), respectively. Among Class I analogs that had no tosyl groups, the most active analogs were the ones with the 4-chlorobenzyl group (**If**, Entry 6, 13.9 μM) and 4-isopropyl benzyl group (**1b**, Entry 2, 13.5 μM). Electron donating methoxy groups on the substituent benzene ring resulted in a decrease in anti-biofilm activity (**Ih**, Entry 8, 70 μM, and **Ii**, Entry 9, 88 μM). Other trends that were noted include the ring type of R_1_ substituent. When comparing furan (**Id**, Entry 4, 84 μM), thiophene (**Ic**, Entry 3, 31 μM) and benzyl (**Ie**, Entry 5, 22 μM) rings in Class I analogs, their activities suggest that the incorporation of five-membered heteroatom-containing rings is not favorable for biofilm inhibition.

**Table 1:** Antibiofilm activity and antibacterial activity of makaluvamine analogs against *S. mutans*.

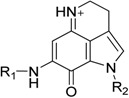					
Entry	Compound No.	R_1_	R_2_	Antibiofilm Activity IC_50_ (μM) ^a^	*S. mutans* MIC_50_ (μM) ^b^
1	Ia	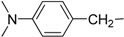	H	38.3 ± 3.6	32.6 ± 0.1
2	Ib	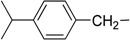	H	13.5 ± 0.8	23.1 ± 0.2
3	Ic	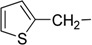	H	30.9 ± 4.6	34.2 ± 0.8
4	Id	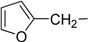	H	84.7 ± 7.5	63.9 ± 13.6
5	Ie	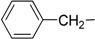	H	22.4 ± 2.6	28.1 ± 0.4
6	If	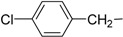	H	13.9 ± 1.7	24.4 ± 0.2
7	Ig	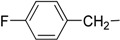	H	30.4 ± 7.6	36.3 ± 0.8
8	Ih	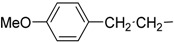	H	70.1 ± 1.0	68.0 ± 4.1
9	Ii	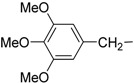	H	88.2 ± 7.6	111.6 ± 27.4
10	IIa	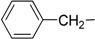	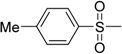	16.3 ± 3.4	26.4 ± 0.6
11	IIb	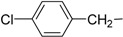	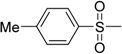	2.5 ± 0.1	22.7 ± 0.6
12	IIc	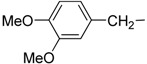	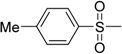	2.6 ± 0.1	28.0 ± 1.9
13	IId	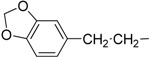	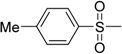	0.4 ± 0.1	1.7 ± 0.1
14	IIe	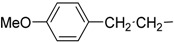	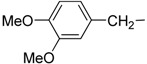	2.0 ± 0.1	5.8 ± 2.8
15	2A4 ^c^	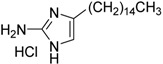		0.94 ± 0.02	2.0 ± 0.5

^a^ Determined by *S. mutans* biofilm inhibition assay; ^b^ determined by *S. mutans* growth inhibition assay; ^c^ reference biofilm inhibitor [34]; ^a,b^ measurements were carried out in triplicate, and the average value with standard deviation is reported.

After having success in identifying makaluvamine analogs as potent biofilm inhibitors, bactericidal activities against *S. mutans* were investigated to test whether the observed biofilm inhibition is a result of bactericidal activity. All makaluvamine analogs were found to be bactericidal against *S. mutans* with MIC_50_ values ranging from 14.7 μM to 67 μM. A comparison of the biofilm inhibition to the analogs’ growth inhibition suggests that the two classes of compounds behave differently. Consistent with their biofilm inhibitions, Class I compounds are less potent bactericidal agents when compared to Class II compounds. The most potent biofilm inhibitor with a free NH on the pyrrole ring was also found to be the one with the most antibacterial activity (**If**, Entry 6). Within this class of compounds, the reported biofilm inhibition directly correlates to the bactericidal activity, leading to the conclusion that the observed anti-biofilm activity of these analogs stems from their ability to kill *S. mutans*.

In contrast, analogs with a tosyl group on pyrrole N (Class II Compounds **IIa**–**e**, Entries 10–14) display bactericidal concentrations an order of magnitude greater than the reported biofilm activity. This suggests that the inhibition of biofilm formation is a more selective process that is not purely through the result of bacterial death.

As stated before, extensive research has been performed on makaluvamine analogs, which classifies them as potent anticancer agents. Their reported mechanism of action as anticancer agents is a result of the inhibition of the enzyme, human topoisomerase II [29,30]. Hence, it is plausible that the antibacterial activity against *S. mutans* investigated could be the result of the inhibition of the bacterial homolog of topoisomerase II, gyrase A. This enzyme is responsible for catalyzing the interconversion of topological isomers of double-stranded DNA rings and for negatively supercoiling DNA [35]. It is crucial for bacterial growth, and thus, its inhibition would result in bacterial death.

## 4. Conclusions

*S. mutans* plays a key role in the formation of dental biofilms. The development of small molecules that inhibit *S. mutans* biofilms may help design anti-caries therapies. In this study, 14 makaluvamine analogs were synthesized and evaluated for their antibacterial activity against *S. mutans* and for their ability to inhibit biofilm formation. All makaluvamine analogs displayed biofilm inhibition with the IC_50_ values ranging from 0.4 μM to 88 μM. The observed bactericidal activities of several makaluvamine analogs were found to be consistent with the anti-biofilm activities, leading to the conclusion that the anti-biofilm activity of these analogs stems from their ability to kill *S. mutans*. However, for the three most active *N*-Tosyl analogs, the biofilm IC_50_ values were at least an order of magnitude lower than that of the bactericidal activities, indicating that the biofilm activity of these analogs is independent of the bactericidal activity, at least in part. These compounds are promising leads to develop biofilm-selective inhibitors. Since the makaluvamine analog’s anti-cancer activity functions through inhibiting DNA topoisomerase II, it is possible that they also inhibit the activity of gyrase A of *S. mutans*, a human topoisomerase II homolog, thereby inhibiting bacterial growth.
